# Supplementary figures and images for: Effects of Sedentary Behavior Interventions on Mental Well-Being and Work Performance While Working from Home during the COVID-19 Pandemic: A Pilot Randomized Controlled Trial
Source: Int J Environ Res Public Health. 2022 May 24;19(11):6401. doi: 10.3390/ijerph19116401 (PMC9180109; doi:10.3390/ijerph19116401)

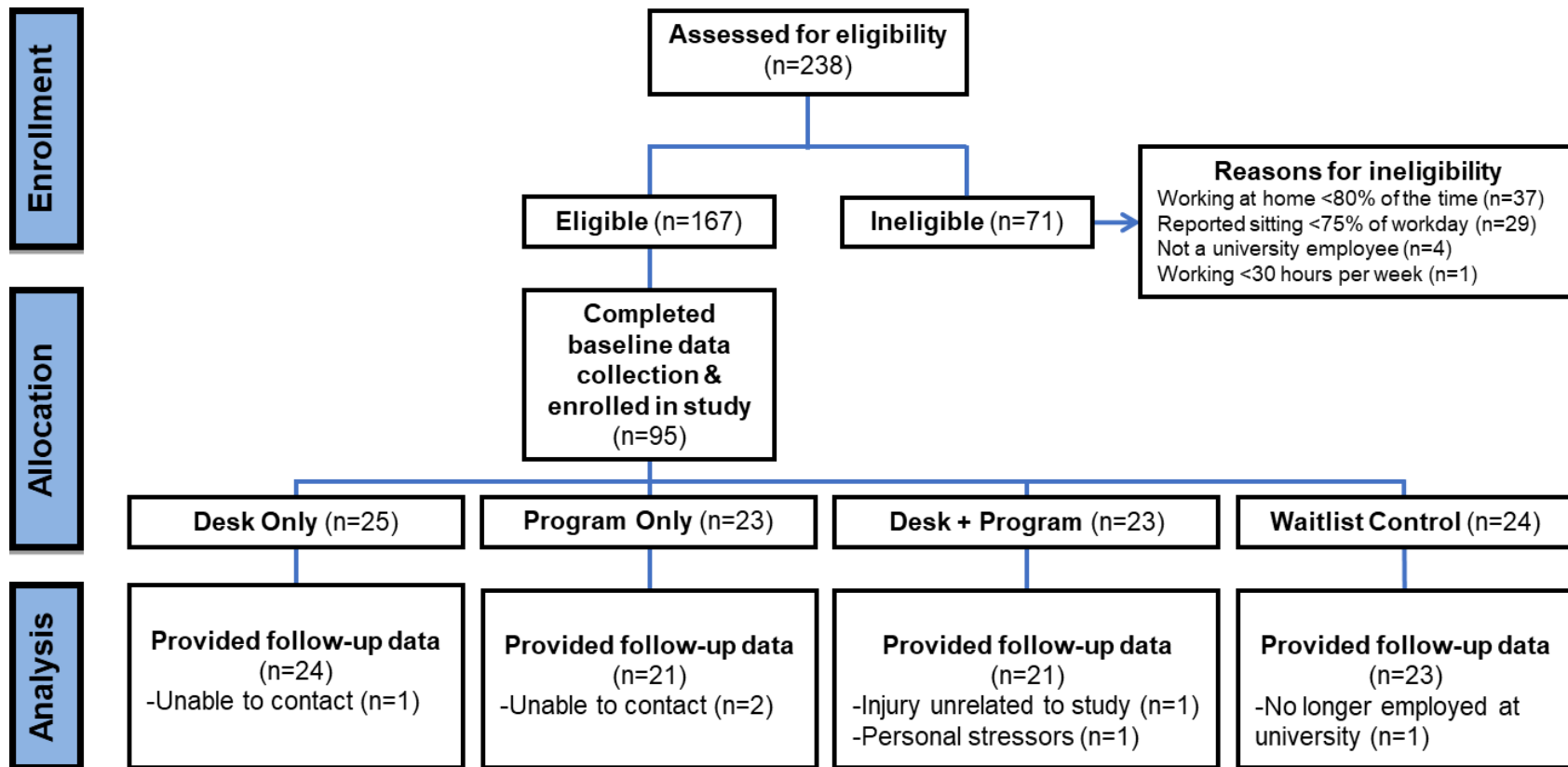

Figure S1. The CONSORT diagram.

Supplement: Supplementary file 1 [file ijerph-19-06401-s001.zip › ijerph-1715728-supplementary.pdf]
